# Identification of a small molecule activator of *SIRT1* gene expression

**DOI:** 10.18632/aging.100539

**Published:** 2013-03-06

**Authors:** Si-Young Cho, Miook Cho, Dae Bang Seo, Sang Jun Lee, Yousin Suh

**Affiliations:** ^1^ R&D Center, Amorepacific Corporation, Gyeonggi-do, Korea 446-729; ^2^ Department of Genetics, Albert Einstein College of Medicine, Bronx NY 10461, USA; ^3^ Department of Medicine, Albert Einstein College of Medicine, Bronx NY 10461, USA; ^4^ Institute of Aging Research, Guangdong Medical College, Dongguan, China

**Keywords:** SIRT1, gene expression, small molecule activator, senescence, syringaresinol

## Abstract

Increased *SIRT1* expression exerts beneficial effects in transgenic animal models, ameliorating the onset and progression of aging-related disease phenotypes in various organs including the heart. The potential beneficial effects of SIRT1 have made SIRT1 a prime therapeutic target for age-related diseases and considerable efforts led to the identification of small molecule activator of SIRT1 protein. Thus far, however, a small molecule activator of *SIRT1* gene expression has not been reported. Here, we report that syringaresinol, isolated from *Panax ginseng* berry pulp, is an activator of *SIRT1* gene expression. Using human umbilical endothelial cells (HUVECs), we show that syringaresinol treatment induced binding of FOXO3 to the *SIRT1* promoter in a sequence-specific manner, leading to induction of *SIRT1* expression. Increased *SIRT1* expression in HUVECs by syringaresinol treatment delayed cellular senescence and improved various markers of endothelial functions in a FOXO3 dependent manner. Collectively, these findings bring to light a new transcription activator of SIRT1 that may have therapeutic potential.

## INTRODUCTION

SIRT1 is a NAD^+^-dependent protein deacetylase that regulates stress response, metabolic homeostasis, and aging in animal models [1]. Tissue-specific overexpression of *SIRT1* was shown to protect mice against age-related disorders such as cardiovascular, neurodegenerative, and metabolic diseases [2, 3], making SIRT1 a prime therapeutic target for such diseases[4-6]. Several small molecule activators of SIRT1 activity have been described and are currently being tested for clinical use against age-related diseases [7-9]. To date, a small molecule activator of *SIRT1* gene expression has not been reported.

## RESULTS AND DISCUSSION

### Discovery of small molecule activator of *SIRT1* gene expression

**Figure 1:**
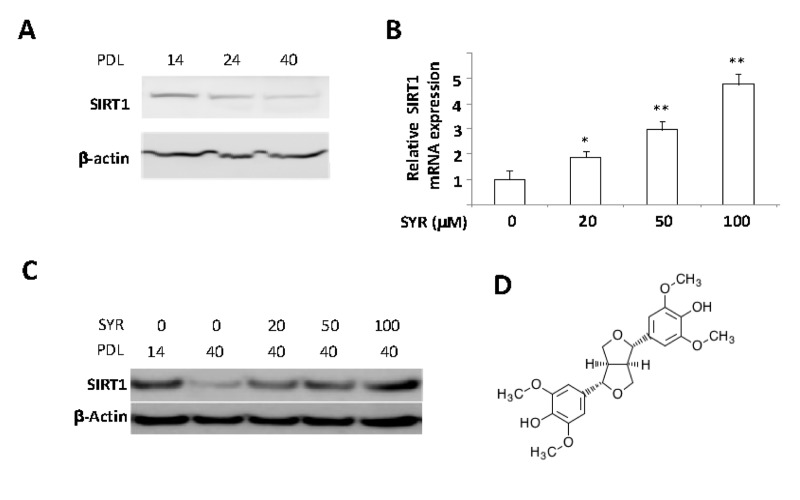
Activation of *SIRT1* gene expression by syringaresinol. (**A**) SIRT1 protein levels were determined by Western blot in PDL 14, 24, and 40 of HUVECs. (**B**) mRNA levels of SIRT1 are measured in PLD 40 HUVECs cultured with various doses of syringaresinol (SYR) from the PDL14. (**C**) SIRT1 protein levels (western blot) at PDL40 in HUVECs treated every 48 hours starting from PDL14 with different doses of syringaresinol. (**D**) Chemical structure of (+)-syringaresinol purified from *Panax ginseng* berry pulp. All the results are either representatives or means ± S.E of at least three independent experiments. Significance was assessed by t-test. *P < 0.05, **P > 0.01.

One of the most prominent effects of increased *SIRT1* expression is protection of endothelial cells against cellular senescence [10]. To identify small molecule transcriptional activators of *SIRT1*, we developed a cell-based assay for increased *SIRT1* gene expression during cellular senescence in human umbilical vein endothelial cells (HUVECs). As previously reported [11], SIRT1 mRNA decreased significantly in HUVECs between population doubling level (PDL) 14 and PDL40 (Figure 1A). We first screened the extracts of various medicinal herbs by culturing HUVECs starting at PDL14 in the presence of each extract in the media until the cells reached PDL40. The extract of *Panax ginseng* berry pulp induced SIRT1 mRNA levels up to 2 fold at PDL40, as compared to untreated controls (Supplemental Figure 1A and 1B). To isolate an active compound responsible for increased *SIRT1* expression during senescence, we performed serial purification steps as described in Supplemental Figure 1C. We identified syringaresinol (4,4’-[(1S,3aβ,6aβ)-Tetrahydro-1H,3H-furo[3,4-c]furan-1β,4β-diyl]bis(2,6-dimethoxyphenol)) as the active compound in*Panax ginseng* berry pulp (Figure 1D), which stimulated expression of both SIRT1 mRNA and protein levels up to five fold (Figure 1B and 1C) without showing any toxicity up to 200μM (Supplemental Figure 1D).

### Identification of a syringaresinol-responsive element in the *SIRT1* gene promoter

**Figure 2:**
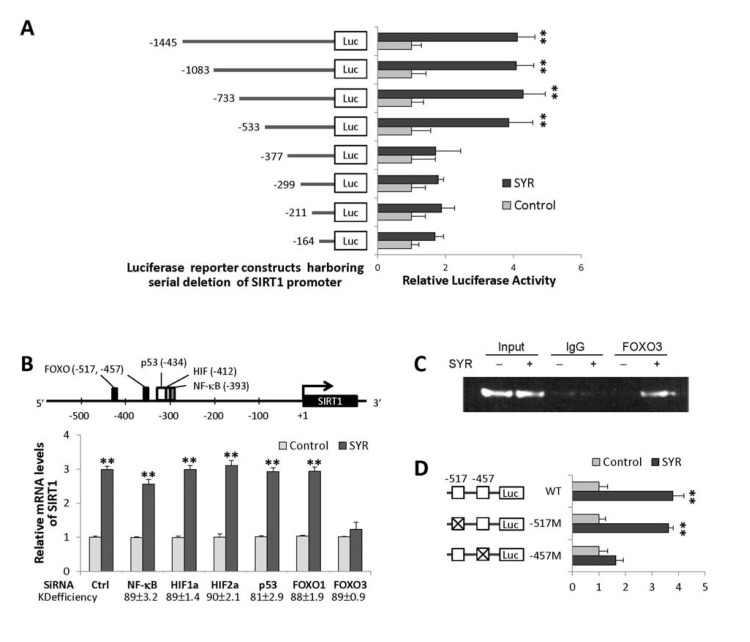
Activation of *SIRT1* gene expression by syringaresinol through FOXO3 binding. (**A**) Relative activities of luciferase expressed from different *SIRT1* promoter constructs in HUVECs at PDL14 treated with (SYR) and without (Control) 50 μM syringaresinol. (**B**) Predicted binding sites for FOXOs, p53, HIFs, and NF-κB in the proximal SIRT1 promoter region, and relative *SIRT1* mRNA levels (qRT-PCR) after knock-down of *NF-*κ*B*, *HIF-1*α, *HIF-1*α, *p53, FOXO1*, and *FOXO3* by gene-specific siRNAs in HUVECs at PDL14 treated with (SYR) and without (Control) 50 μM syringaresinol. The knock-down (KD) efficiency (%) of each is indicated. (**C**) Binding of FOXO3 (chromatin immunoprecipitation followed by qPCR: qChiP) to SIRT1 promoter region (−533 to −352) in HUVECs at PDL14 treated with (+) and without (−) 50 μM syringaresinol. (**D**) Relative luciferase activities of *SIRT1* promoter constructs harboring site-specific changes (indicated as X) in each (−517 or −457) of predicted FOXO3 binding sites in HUVECs at PDL14 treated with (SYR) and without (Control) 50 μM syringa-resinol. All the results are either representatives or means ± S.E of at least three independent experiments. Significance was assessed by *t*-test. **P < 0.01.

We next performed luciferase transcription reporter assays to identify regions of the *SIRT1* promoter required for induction. We found that a syringaresinol-responsive element resided between −377 and −533 bp from the*SIRT1* transcription initiation site (Figure 2A). *In silico* analysis predicted that this region contains canonical binding sites of FOXOs, p53, NF-κB, and HIFs (Figure 2B). Knock down of FOXO3, but not the other transcription factors, abrogated *SIRT1* induction in response to syringaresinol, and FOXO3 binds to the syringaresinol-responsive region of *SIRT1* promoter (Figure 2B and 2C). Site-directed mutagenesis analysis indicated that, of the two predicted FOXO binding sites (−517 and −457), the -457 site was required for the upregulation of *SIRT1* by syringaresinol (Figure 2D).

### Delayed cellular senescence and enhanced endothelial functions by syringaresinol

**Figure 3:**
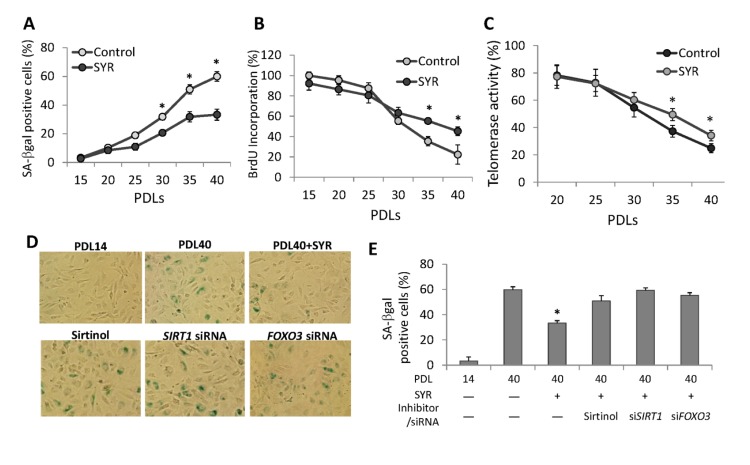
Effects of syringaresinol treatment on senescence. (**A**) Quantification of senescence by using SA-*β*-Galstaining for cells cultured from PD14 to PDL40 with or without 50μM syringaresinol. (**B**) Measurement of proliferative capacity of cells cultured with or without syringaresinol (50μM) was measured using BrdU incorporation. (**C**) Telomerase activities were measured in HUVECs at different PDLs with or without syringaresinol and the relative levels to the activity at PDL 14 were compared. (**D-E**) SA-*β*-Gal staining of HUVECs and quantifications of SA-*β*-Gal positive cells at PDL40 treated every 48 hours starting from PDL14 with 50μMsyringaresinol (SYR), 10mM Sirtinol, *SIRT1* siRNA, and *FOXO3* siRNA. All the results are either representatives or means ± S.E of at least three independent experiments. Significance was assessed by *t-*test. *P < 0.05.

**Figure 4:**
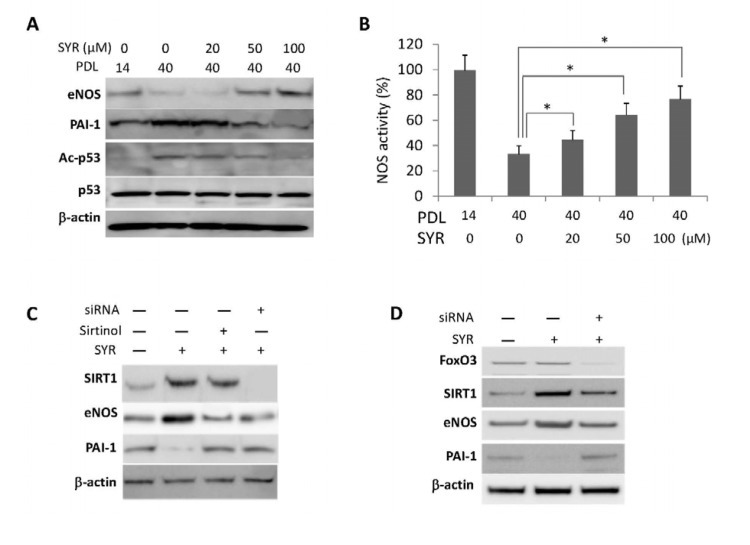
Effects of syringaresinol treatment on endothelial functions. (**A**) Protein levels of eNOS, PAI-1, and acetylation of p53 (Ac-p53) at lysine 373/382 in HUVECs at PDL40 treated every 48 hours starting from PDL14 with different doses of syringaresinol (SYR). (**B**) NOS activities measured in HUVEC cultured in different doses of syringaresinol. (**C-D**) Protein levels of SIRT1, eNOS and PAI-1 in HUVECs at PDL14 treated with siRNAs against SIRT1 (**C**) and FOXO3 (**D**). All the results are either representatives or means ± S.E of at least three independent experiments. Significance was assessed by *t*-test. *P < 0.05.

Senescence of HUVECs can be detected by changes in cellular morphology and senescence-associated beta-galactosidase (SA-*β*-Gal) staining at PDL40 (Figure 3D). We found that cells grown in media supplemented with syringaresinol showed delayed senescence as measured by SA-*β*-Gal (Figure 3A, 3D, and 3E) and other molecular markers of senescence (Supplemental Figure 2), increased proliferative capacity (Figure 3B), and increased telomerase activity (Figure 3C) at PDL40 as compared to non-treated cells. These effects were prevented by the SIRT1 inhibitor Sirtinol as well as by *SIRT1* siRNA (Figure 3D and 3E), suggesting that they resulted from activation of *SIRT1* expression. Knock-down of FOXO3 also abrogated the effects of syringa-resinol, consistent with the model that the compound induces*SIRT1* expression in a FOXO3-dependent manner (Figure 3D and 3E). The atheroprotective effects of SIRT1 are thought to involve inhibition of apoptosis and promotion of vasodilation [10, 12], and mediated in part by activation of endothelial nitric oxide synthase (eNOS) [13]. We investigated the effects of syringaresinol treatment on endothelial function by assessing levels of eNOS and plasminogen activator inhibitor-1 (PAI-1), and NOS activity. We also measured the levels of p53 acetylation as a marker of SIRT1 activation. Syringaresinol treatment decreased the acetylation of p53 (Lys373/382) and levels of PAI-1, whereas it increased the eNOS levels and NOS activity in HUVECs at PDL 40 (Figure 4A, 4B and Supplemental Figure 3). Knock-down of either *SIRT1* or *FOXO3* by siRNA abolished the effects of syringaresinol treatment on the markers of endothelial functions (Figure 4C and 4D), suggesting that these beneficial effects were mediated through a SIRT1- and FOXO3-dependent manner.

In summary, we report discovery of the first small molecule activator of *SIRT1* gene expression. Increased expression of *SIRT1* by syringaresinol is mediated by FOXO3 and resulted in delayed cellular senescence and enhanced endothelial function, suggesting a possible utility of the compound in therapeutic intervention of age-related diseases.

## MATERIALS AND METHODS

### Cell culture

Human umbilical vein endothelial cells (HUVECs) were purchased from LONZA (Walkersville, MD, USA) and cultured in endothelial growth medium (EGM-2, EGM-2 SingleQuots, LONZA Walkersville, Inc., Walkersville, MD, USA) with 100 units/ml penicilli and 100 μg/ml streptomycin in a humidified atmosphere of 5% CO_2_. When cells reached 80% confluence, they were counted and seeded with 6×10^5^ cells per100-mm gelatin-coated dish. The rate of PDL was calculated at each passage until growth arrest based on the following formula: PDL= (log_10_Y-log_10_X)/log_10_2 (Y indicates the number of cells counted at the end of the passage; X is the number of cells seeded). The entity of HUVECs was demonstrated by staining of endothelial specific marker von Willebrand factor at PDL40.

### Extraction, separation, and treatment of herbal extracts to HUVECs

To identify transcriptional activator of *SIRT1*, we selected 12 natural products that had traditionally been used as a health food for anti-aging in East Asia. Twelve medicinal herbs were extracted by heating at 80°C in 70% ethanol for 3 h. The ethanol extract was dried under vacuum, dissolved in distilled water and filtered with Whatman no. 1 filter paper (Maidstone, UK). The filtered solution was heat-treated, condensed by an evaporator, and then refrigerated. HUVECs were treated with each extract at 200 μg/ml, which was added every 48 h starting from population doubling level 14 (PDL14) untilPDL 40. At PDL 40, SIRT1 mRNA and protein levels were examined.

### Isolation of bioactive chemical compound from the *Panax ginseng* berry

To obtain the effective fractions of *Panax ginseng* berry extracts that induced *SIRT1* expression, the seeds were removed, and the pulp was collected and treated with 70% ethanol. After mixing for 20 min, the mixture was allowed to stand at 4 °C for 24 hours. After filtering the mixture, the residue was extracted twice again using 70% ethanol. The ethanol extract was dried under vacuum, dissolved in distilled water, and filtered with Whatman no. 1 filter paper (Maidstone, UK). The filtered solution was treated with water-saturated butanol. The butanol-soluble fraction (194 g) was chromatographed on reverse-phase flash column eluted with a step-wise gradient of metanol to yield ten fractions [1-10]. Fraction 3 was chromatographed on sephadex L-20 column eluted with 50% aqueous methanol to yield eight subfractions (3A-H). Bioactive compound (13 mg) was obtained from fraction 3G (300 mg) using a preparative silica gel TLC eluted with chroloform-metanol (10:1, v/v). The physico-chemical data of this compound are as follows: (+)-syringaresinol (C_22_H_26_O_8_): [α]_D_ +40.9° (c0.1, in CHCl_3_); ESI-MS: m/z 440.9 [M+Na]^+^, 858.9 [2M+Na]^+^. Syringaresinol was dissolved in DMSO to make a stock solution.

### Senescence-associated β-galactosidase (SA-*β*-Gal)

HUVECs at PDL40 were grown in media supplemented with 50 μMsyringaresinol or vehicle (DMSO) (added every 48 hours)from PDL14. SA-*β*-Gal staining was performed using Cellular Senescence Assay kit (Cell Biolabs, Inc., San Diego, CA, USA) according to the manufacturer's instruction. Briefly, cells were washed with PBS and fixed for five minutes at room temperature. Cells were then kept in Staining Working Solution at 37 °C for 16 hours. After washing with PBS, 20% glycerol solution was overlaid and cells counted under a microscope. The absolute number of blue cells was counted out of 2000 cells.

### Cell proliferation, telomerase activity

Cell proliferation analysis was performed using Cell Proliferation ELISA (colorimetric) and BrdU incorporation assay (Roche Applied Science, Indianapolis, IN, USA), according to the manufacturer'sprotocol. Cell proliferation was monitored at 48 hours of each passage. HUVECs (2×10^5^ cells) were washed in PBS and the pellet was lysed with 30 μl of lysis reagent for 30 minutes at 4°C. Then, proteins were centrifuged for 20 minutes at 10.000 × g and the concentrations were determined using the supernatant using the Bradford assay (Bio-Rad, Hercules, CA, USA). Telomerase activity was measured in 2 μg proteins by the Telo TAGGG Telomerase PCR ELISA PLUS Kit according to the protocol form the manufacturer (Roche Applied Science, Indianapolis, IN, USA).

### NOS activity assays

NOS activity was determined using a NOS assay kit (Calbiochem, Merck, Damstadt, Germany) according to the manufacturer's instructions.

### Inhibition of SIRT1 activity and knock down of *SIRT1* gene

To inhibit the function of SIRT1, HUVECs were treated with 10 mM Sirtinol (Calbiochem, San Diego, CA, USA) or *SIRT1* siRNA. Validated Stealth siRNA for *SIRT1* (5’-GCAACAGCAUCUUGCCUGAUUUGUA- 3’, nucleotides 1152-1175 of human SIRT1 mRNA) and the appropriate control RNAi were purchased from Invitrogen (Carlsbad, CA, USA). Transient transfection of siRNA into HUVECs were done using lipofectaminutes RNAi MAX (Invitrogen).

### Plasmid constructs and site-directed mutagenesis

The human *SIRT1* promoter region (-1455/-1), was amplified by PCR from HUVEC genomic DNA by using Phusion high fidelity DNA polymerase (New England Biolabs MA, USA). The 5' and 3' amplification primers included *Mlu*I and *Xho*I restriction sites, respectively. The amplified products were ligated into the *Mlu*I and *Xho*I sites of pGL3 basic (Promega, Madison, WI, USA), followed by sequencing confirmation. The clone was named pGL-*SIRT1*-1455. Luciferase reporters containingvarious sizes of *SIRT1* promoter (1083, 733, 553, 377, 299, 211, and 164 bp) were constructed by inserting PCR-amplified DNA fragments into pGL3 basic. The constructs are named after the size of promoter inserted; pGL-*SIRT1*-1083, pGL-*SIRT1*-733, and pGL-*SIRT1*-553, pGL-*SIRT1*-377, pGL-*SIRT1*-299, pGL-*SIRT1*-211, pGL-*SIRT1*-164. The primer sequences used for amplification are described in Table S1.

Site-directed mutagenesis of putative FOXO binding sites was performed using a QuikChange site-directed mutagenesis kit (Stratagene, La Jolla, CA, USA). The oligonucleotides used for mutagenesis are listed in the Supplemental table 1.

### Luciferase reporter assays

HUVECs in 24-well plates were transfected with series of reporter plasmids containing the *SIRT1* promoter or adenine substitutions (720 ng) with pRL-TK (80ng, Promega, Madison,WI,USA)) with a 10:1 ratio. Transfections were performed with TransPass™ HUVEC Transfection Reagent (New England Biolabs, Inc, Ipswich, MA, USA). At 24 hours after transfection, the cells was washed with PBS, stimulated syringaresinol (50 μM) for 24 h, and lysed in Passive Lysis Buffer (Promega). Then, luciferase activities were measured using a Dual-Luciferase Reporter Assay System (Promega) on a microplate luminometer (SpectraMax, Molecular Devices, Sunnyvale, CA, USA). Promoter activities were normalized with Renilla luciferase activity, and expressed as a ratio relative to the firefly luciferase activity of -1500 plasmid-trasnsfected cells incubated without syringaresinol.

### siRNA treatment

HUVECs were transfected using DharmaFECT 4 (Dharmacon, Thermo Fisher Scientific Inc., CO, USA) with 100 nM ON-TARGETplus SMARTpool for FOXO1, FOXO3, p53, HIF-1α, HIF-2α, and NF-kB, (Dharmacon). After 24 hours, cells were washed with PBS, treated with syringaresinol for 24hours and harvested for mRNA analysis. Knock-down efficiency of each gene was at least 80% as measured by qRT-PCR analysis.

### Chromatin Immunoprecipitation (ChIP) assay

The ChIP was done following the manufacturer's instructions (Upstate, CS, USA). HUVECs were treated for 24 h with or without 50 μM syrinaresinol. The cells were fixed with 1% formaldehyde at 37 °C for 10 minutes, lysed, and sonicated. Soluble chromatins were immune-precipitated with anti-FOXO3 antiserum (Santa Cruz, CA, USA) or an equal amount of rabbit IgG (Santa Cruz, CA, USA). After dissociation and purification of DNAs from proteins, 1% input and immunoprecipitated DNA samples were subjected to PCR using a pair of primers flanking the FoxO3a-binding site (sense 5’-CTCTTCCTACTTATTAACAA-3’ and antisense 5’-CGGAACAGCTCAAGTTTTGG-3’) that amplify a 180-bp product size. Standard PCRs were performed.

## SUPPLEMENTAL DATA



## References

[R1] Nakagawa T, Guarente L (2011). Sirtuins at a glance. J Cell Sci.

[R2] Guarente L, Franklin H (2011). Epstein Lecture: Sirtuins, aging, and medicine. N Engl J Med.

[R3] Herranz D, Serrano M (2010). Impact of Sirt1 on mammalian aging. Aging (Albany NY).

[R4] Hung CW, Chen YC, Hsieh WL, Chiou SH, Kao CL (2010). Ageing and neurodegenerative diseases. Ageing Res Rev.

[R5] Haigis MC, Sinclair DA (2010). Mammalian sirtuins: biological insights and disease relevance. Annu Rev Pathol.

[R6] Sundaresan NR, Pillai VB, Gupta MP (2011). Emerging roles of SIRT1 deacetylase in regulating cardiomyocyte survival and hypertrophy. J Mol Cell Cardiol.

[R7] Lavu S, Boss O, Elliott PJ, Lambert PD (2008). Sirtuins--novel therapeutic targets to treat age-associated diseases. Nat Rev Drug Discov.

[R8] Milne JC, Denu JM (2008). The Sirtuin family: therapeutic targets to treat diseases of aging. Curr Opin Chem Biol.

[R9] Vetterli L, Maechler P (2011). Resveratrol-activated SIRT1 in liver and pancreatic beta-cells: a Janus head looking to the same direction of metabolic homeostasis. Aging (Albany NY).

[R10] Zu Y, Liu L, Lee MY, Xu C, Liang Y, Man RY, Vanhoutte PM, Wang Y (2010). SIRT1 promotes proliferation and prevents senescence through targeting LKB1 in primary porcine aortic endothelial cells. Circ Res.

[R11] Ota H, Akishita M, Eto M, Iijima K, Kaneki M, Ouchi Y (2007). Sirt1 modulates premature senescence-like phenotype in human endothelial cells. J Mol Cell Cardiol.

[R12] Zhang QJ, Wang Z, Chen HZ, Zhou S, Zheng W, Liu G, Wei YS, Cai H, Liu DP, Liang CC (2008). Endothelium-specific overexpression of class III deacetylase SIRT1 decreases atherosclerosis in apolipoprotein E-deficient mice. Cardiovasc Res.

[R13] Mattagajasingh I, Kim CS, Naqvi A, Yamamori T, Hoffman TA, Jung SB, DeRicco J, Kasuno K, Irani K (2007). SIRT1 promotes endothelium-dependent vascular relaxation by activating endothelial nitric oxide synthase. Proc Natl Acad Sci U S A.

