# Supplementary material for: Identification of a small molecule activator of SIRT1 gene expression
Source: Aging (Albany NY). 2013 Mar 6;5(3):174–82. doi: 10.18632/aging.100539 (PMC3629289; doi:10.18632/aging.100539)
Supplement: Supplementary file 1 [file aging-05-174-s001.pdf]

apolipoprotein E-deficient mice. Cardiovasc Res. 2008; 80:191-199.

13. Mattagajasingh I, Kim CS, Naqvi A, Yamamori T, Hoffman TA, Jung SB, DeRicco J, Kasuno K and Irani K. SIRT1 promotes endothelium-dependent vascular relaxation by activating endothelial nitric oxide synthase. Proc Natl Acad Sci U S A. 2007; 104:14855-14860.

SUPPLEMENTAL DATA

Supplemental Table 1. Primers used for generation of luciferase reporter constructs with various sizes of the *SIRT1* promoter and mutagenesis

Amplicon	Primer sequence
SIRT1 promoter cloning	
1596bp Forward	5'-CATCACGCGTGTTCTTAACCAGTGGTAGACCAGAA-3'
1083bp Forward	5'-TTCAGGGAGA GAGGAAAGTG GAAG-3'
773bp Forward	5'-TTTGAAGCCA AGCTGGGGCC AGAA-3'
553bp Forward	5'-GGAGCCGCCT CCTTTTGCCT CTCT-3'
377bp Forward	5'-CATCACGCGTCGGACCAAAACTTGAGCTGT
299bp Forward	5'-CATCACGCGTCGAATTTGGCTGCACTACAC
211bp Forward	5'-CATCACGCGTAGACGCAACAGCCTCCGCC
164bp Forward	5'-CATCACGCGTGGCCCGCGTGGGTGGCGGG
All sizes' Reverse	5'-CATGCTCGAGCTTCCAACTGCCTCTCTGGC
FOXO3 site directed mutagenesis	
Site1 Forward	5'-CTCTTCCTACTTATTAACGGGACAGAACGACTATCCAACGTATTTCAGGG-3'
Site1 Reverse	5'-CCCTGAAATACGTTGGATAGTCGTTCTGTCCCGTTAATAAGTAGGAAGAG-3'
Site2 Forward	5'-TAGCCAGCTTCAGCTGTGCCCTAACCCTTAGCTAAATATAGACAAGGCTA-3'
Site2 Reverse	5'-TAGCCTTGTCTATATTTAGGCTAAGGGTTAGGGCACAGCTGAAGCTGGCTA-3'

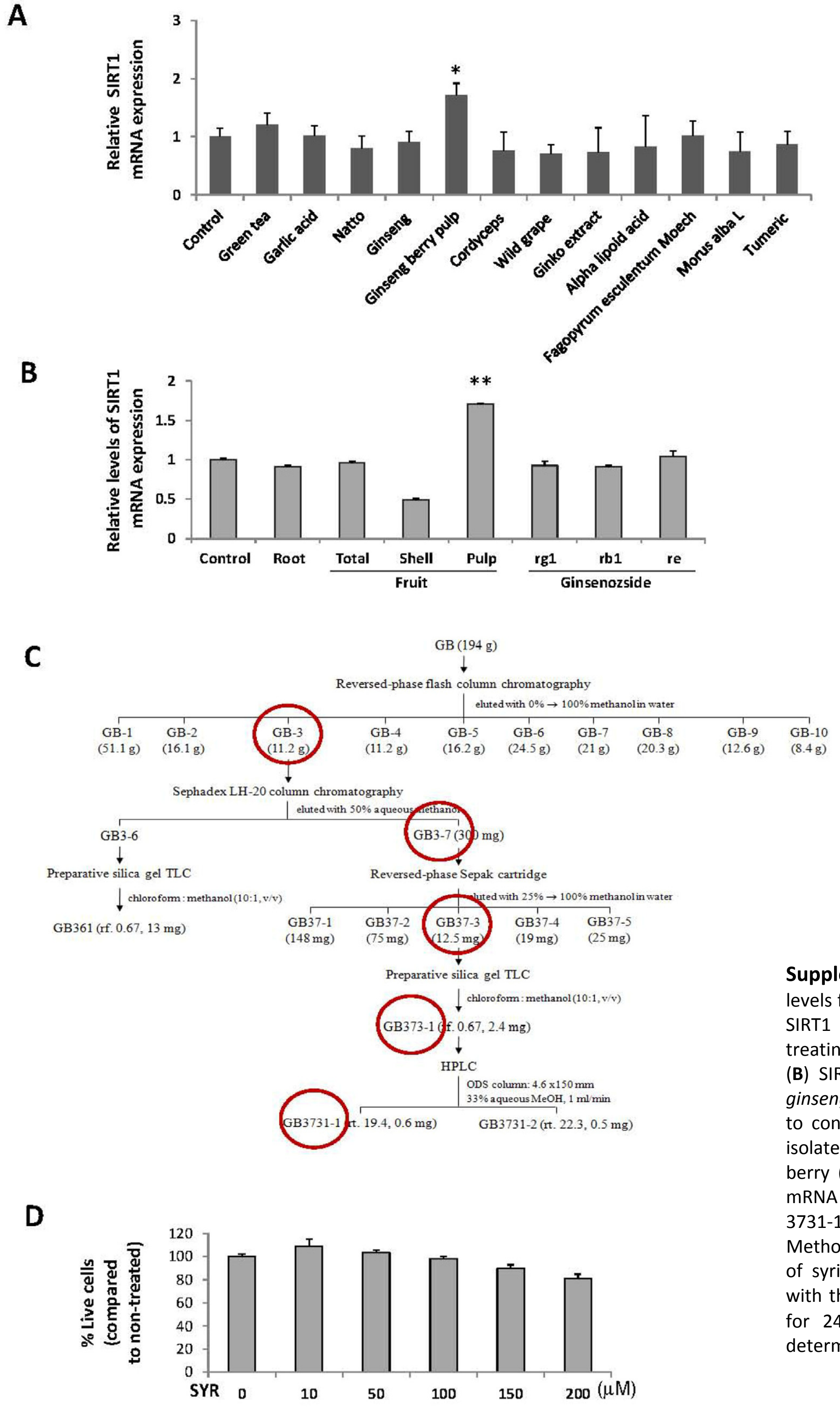

Supplemental Figure 1. Measurement of SIRT1 levels for identification of *SIRT1* gene activator. (**A**) SIRT1 mRNA levels measured by qRT-PCR after treating HUVECs with the extracts from 12 herbs. (**B**) SIRT1 mRNA levels in various part of *Panax ginseng* were measured by qRT-PCR and compared to control (non-treated). (**C**) Purification steps to isolate a bio-active compound, from *Panax ginseng* berry (GB) extracts. Fractions that induced SIRT1 mRNA levels in HUVECs are indicated in circles. GB-3731-1 was found to be syringaresinol. See Methods for detailed information. (**D**) Cytotoxicity of syringaresinol was tested by treating HUVECs with the indicated concentration of syringaresinol for 24 h. Data are mean ± S.E. of triplicate determinations.

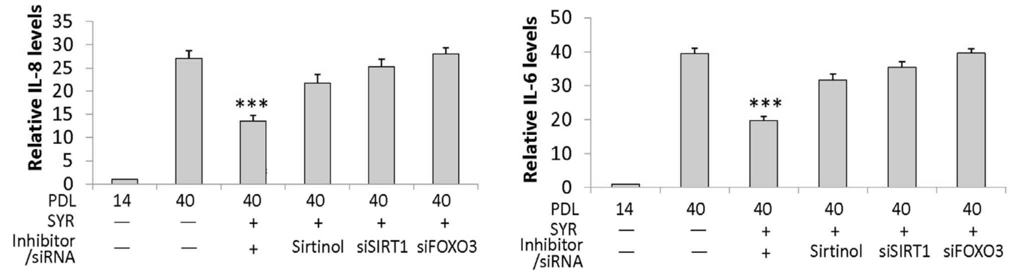

Supplemental Figure 2. IL-6 and IL-8 produced by cells with indicated treatment were determined by ELISA and the relative levels to PDL14 were compared. The results are mean ± S.E of four independent experiments and significance was assessed by *t*-test. *** P < 0.005.

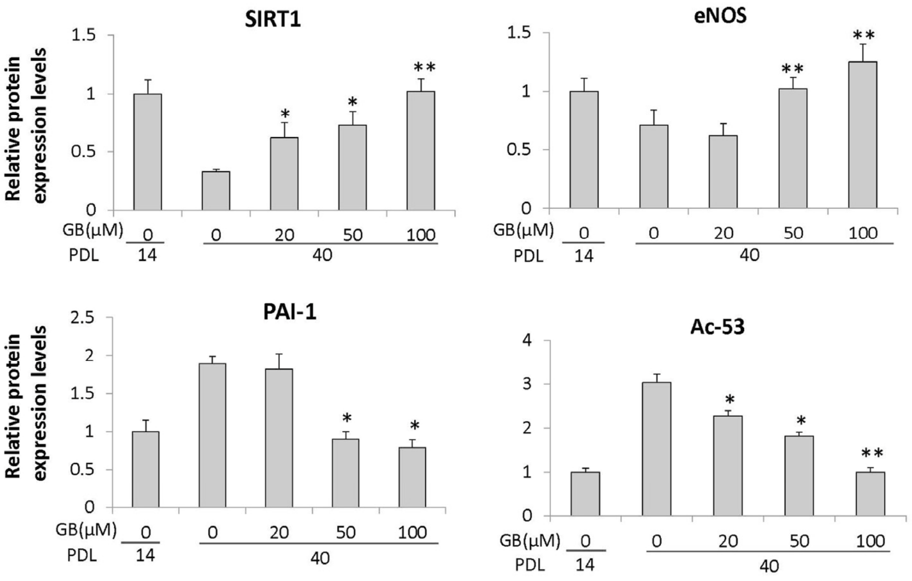

Supplemental Figure 3. Quantification of the protein levels shown in the Fig 4. The results are means ± S.E of three independent experiments. Significance was assessed by *t*-test. *P < 0.05, **P < 0.01.
